# Supplementary material for: Identification of photoperiod-induced specific miRNAs in the adrenal glands of Sunite sheep (Ovis aries)
Source: Front Vet Sci. 2022 Jul 22;9:888207. doi: 10.3389/fvets.2022.888207 (PMC9354845; doi:10.3389/fvets.2022.888207)
Supplement: Supplementary file 1 [file Data_Sheet_1.ZIP › Supplementary file/miRDeep2 file.zip!/miRDeep2 file/Know_Structure/SPLP42A3/oar-mir-127.pdf]

miRBase precursor : oar-mir-127
Total read count : 61139
oar-miR-127 read count : 59839
remaining reads : 1300

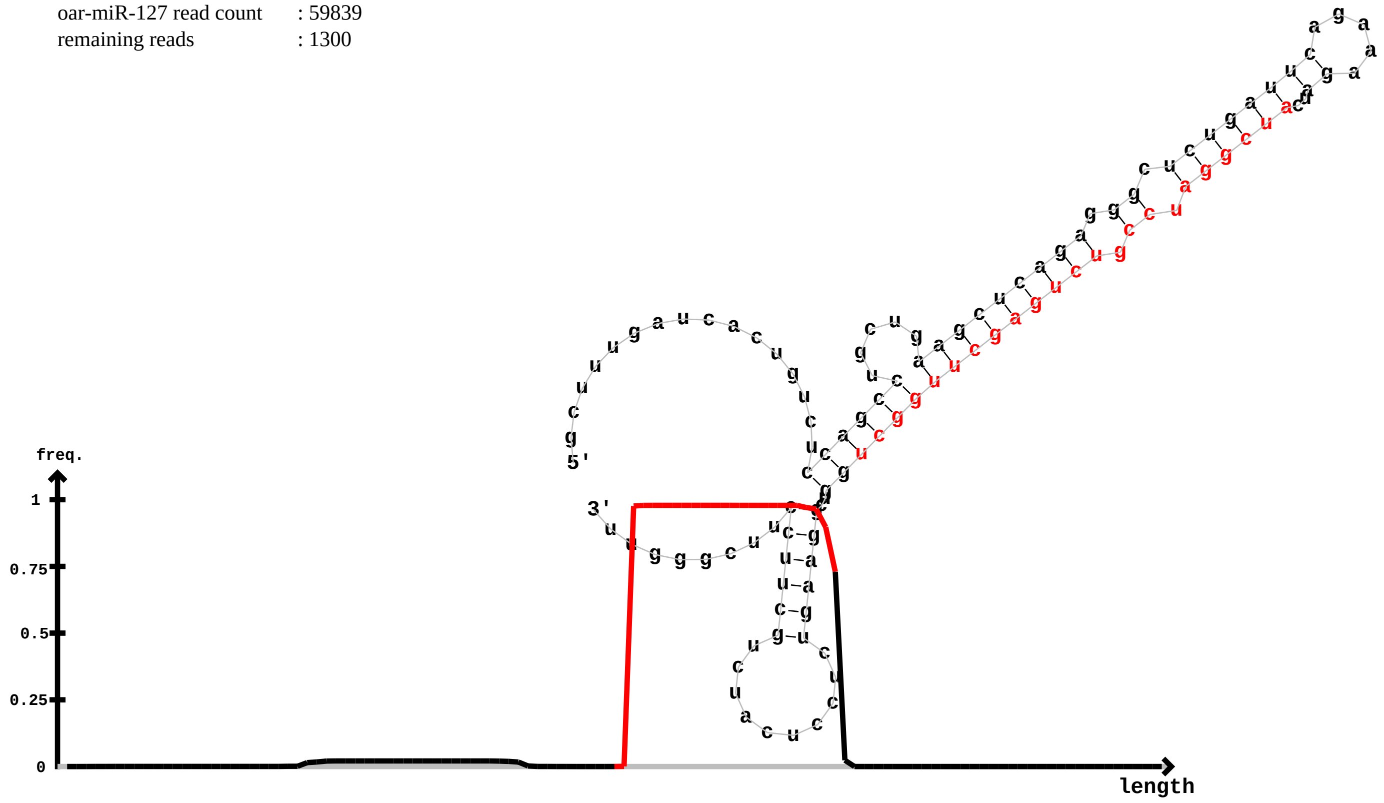

oar-miR-127

5'- gcuuugaucacugucuccagccugcugaagcucagagggcucugauucagaaagaucaucggauccgucugagcuuggcuggucggaagucuccucaucugcuuccucggguu -3'	exp		
................((((((.....(((((((((.((.((((((((.....))..)))))).)).)))))))))))))))..((((((..........)))))).......	reads	mm	sample
gcuuugaucacugucuccagccug...	1	0	seq
.cuuugaucacugucuccagccug...	5	0	seq
..uuugaucacugucuccagccug...	10	0	seq
...uugaucacugucuccagccug...	4	0	seq
....ugaucacugucuccagccu..	1	0	seq
....ugaucacugCcuccagccug...	1	1	seq
....ugaucacugucuccagccug...	5	0	seq
......................ugcugaagcucagagggcuc...	1	0	seq
......................ugcugaagcucagagggcucu..	1	0	seq
......................ugcugaagcucagagggcucug...	12	0	seq
......................ugcugaagcucagagggcucuga..	9	0	seq
......................ugcugaagcucagagggcucugau...	4	0	seq
......................ugcugaagcucagagggcucugauu..	6	0	seq
.......................gcugaagcucagagggcucug...	2	0	seq
.......................gcugaagcucagagggcucuga..	5	0	seq
.......................gcugaagcucagagggcucugau...	2	0	seq
.......................gcugaagcucagagggcucugauu..	2	0	seq
........................cugaagcucagagggcu..	1	0	seq
........................cugaagcucagagggcucug...	12	0	seq
........................cugaagcucagagggcucuga..	48	0	seq
........................cugaagcucagagggcucugau...	138	0	seq
........................cugaagcucagagggcucugauu..	624	0	seq
........................cugaagcucagagggcucugauuc...	29	0	seq
........................cugaagcucagagggcucugauucagaa...	2	0	seq
........................cugaagcucagagggcucugauucagaaagauc..	1	0	seq
.........................ugaagcucagagggcucug...	2	0	seq
.........................ugaagcucagagggcucuga..	1	0	seq
.........................ugaagcucagagggcucugau...	5	0	seq
.........................ugaagcucagagggcucugauu..	133	0	seq
.........................ugaagcucagagggcucugauuc...	7	0	seq
..........................gaagcucagagggcucugau...	1	0	seq
..........................gaagcucagagggcucugauu..	114	0	seq
..........................gaagcucagagggcucugauuc...	82	0	seq
..........................gaagcucagagggcucugauuca..	2	0	seq
..........................gaagcucagagggcucugauucag...	2	0	seq

oar-miR-127

```
gcuuugaucacugucuccagccugcugaagcucagagggcucugauucagaaagaucaucggauccgucugagcuuggcuggucggaagucuccucaucugcuuccuucggguu
...........................aagcucagagggcucugau....................................................................	1	0	seq
...........................aagcucagagggcucugauu...................................................................	12	0	seq
...........................aagcucagagggcucugauuc..................................................................	1	0	seq
...........................aagcucagagggcucugauucag................................................................	5	0	seq
...........................aagcucagagggcucugauucaga...............................................................	5	0	seq
..............................................ucagaaagaucaucggauccguc.............................................	1	0	seq
........................................................caucggauccgucugagcuugg....................................	2	0	seq
.........................................................aucggauccgucugagcuu......................................	2	0	seq
.........................................................aucggauccgucugagcuugg....................................	17	0	seq
.........................................................aucggauccgucugagcuuggc...................................	17	0	seq
.........................................................aucggauccgucugagcuuggcu..................................	1	0	seq
..........................................................ucggauccgucugagcu.......................................	2	0	seq
..........................................................ucggauccgucugagcuu......................................	500	0	seq
..........................................................ucggauccgucugagcuug.....................................	331	0	seq
..........................................................ucggauccgucugagcuugg....................................	4120	0	seq
..........................................................ucggauccgucugagcuuggc...................................	10164	0	seq
..........................................................ucggauccgucugagcuuggcu..................................	43110	0	seq
..........................................................ucggauccgucugagcuuggcug.................................	1418	0	seq
..........................................................ucggauccgucugagcuuggcugg................................	5	0	seq
...........................................................cggauccgucugagcuu......................................	1	0	seq
...........................................................cggauccgucugagcuug.....................................	1	0	seq
...........................................................cggauccgucugagcuugg....................................	10	0	seq
...........................................................cggauccgucugagcuuggc...................................	47	0	seq
...........................................................cggauccgucugagcuuggcu..................................	65	0	seq
...........................................................cggauccgucugagcuuggcug.................................	19	0	seq
............................................................ggauccgucugagcuug.....................................	1	0	seq
............................................................ggauccgucugagcuuggcu..................................	3	0	seq
............................................................ggauccgucugagcuuggcug.................................	1	0	seq
............................................................ggauccgucugagcuuggcugg................................	1	0	seq
............................................................ggauccgucugagcuuggcuggu...............................	1	0	seq
```
